# Contrast-enhanced ultrasound findings of hepatic pseudolipoma: a case report

**DOI:** 10.3389/fonc.2025.1581523

**Published:** 2025-08-01

**Authors:** Luyao Jiang, Zhiqiang Yuan, Yan Luo

**Affiliations:** Department of Medical Ultrasound, West China Hospital, Sichuan University, Chengdu, China

**Keywords:** hepatic pseudolipoma, solitary necrotic nodule, contrast-enhanced ultrasound, benign lesions, case report

## Abstract

**Background:**

Hepatic pseudolipoma is a relatively rare benign tumor of the liver in primary hepatic lesions and is characterized by focal fat around the liver capsule. It typically affects older adult men. In this case report, we discuss the case of a 71-year-old male who presented to our hospital with rectal adenocarcinoma. A pseudolipoma was incidentally detected on a preoperative computed tomography scan of the abdomen. We performed contrast-enhanced ultrasound to clarify the nature of this tumor. To the best of our knowledge, there have not been any documented reports on contrast-enhanced ultrasound findings of hepatic pseudolipoma.

**Findings:**

Hepatic pseudolipoma appears on contrast-enhanced ultrasound as slightly hyperechoic nodule with poorly defined borders and regular morphology.

**Diagnosis:**

On the basis of the patient’s clinical symptoms, test results, and imaging manifestations, the lesion was considered a pseudolipoma, not a liver metastasis from rectal cancer. Pseudolipoma usually does not need treatment.

**Conclusion:**

The current report will help enhance the current knowledge regarding identifying hepatic pseudolipoma by contrast-enhanced ultrasound imaging and review the related literature.

## Introduction

Pseudolipoma, generally defined as pseudolipoma of the Glisson’s capsule, is a mass of fat tissue encapsulated and enveloped by the liver (1). It comes from a region between the liver and the diaphragm of the epiploic appendix that calcifies and then degenerates, allowing vascular supply through the hepatic envelope. It is an extremely rare benign lesion with an incidence of 0.2% (2). Transcutaneous liver biopsy or traumatic fat inclusion within the liver capsule during surgery are potential additional reasons. However, there is no evidence of an association with factors such as surgery, obesity, and alcohol abuse (3). On computed tomography (CT) images, pseudolipoma presents as a well-circumscribed low-density nodule on the surface of the liver with a central attenuation of fat or soft tissue. The diagnosis is usually confirmed by CT (1). Treatment is rarely recommended for hepatic pseudolipoma, as it is generally asymptomatic (4).

## Case presentation

**Figure 1:**
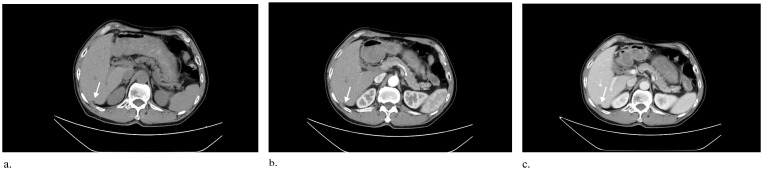
**(a)** Axial unenhanced CT image shows a 1.2x0.8 cm ovoid fat density nodule under the pericardium of the right posterior lobe of the liver (arrow). **(b, c)** Axial contrast-enhanced CT scans of the arterial and portal venous phases show no enhancement of the fat-dense nodule (arrows), and the nodule is accompanied by peripheral calcification in the portal venous phase.

**Figure 2:**
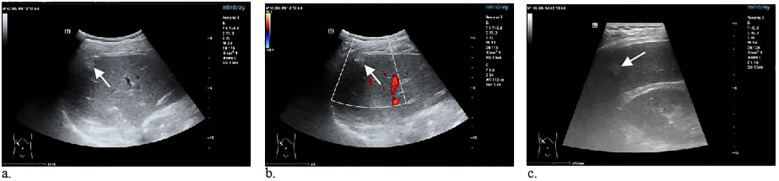
**(a, b)** Greyscale ultrasound (US) image shows a slightly hyperechoic nodule under the pericardium with poorly defined borders and regular morphology. Color Doppler US image shows no significant blood flow signal(arrows). **(c)** High-frequency US image shows a slightly hyperechoic nodule in the right posterior lobe of the liver(arrow).

**Figure 3:**
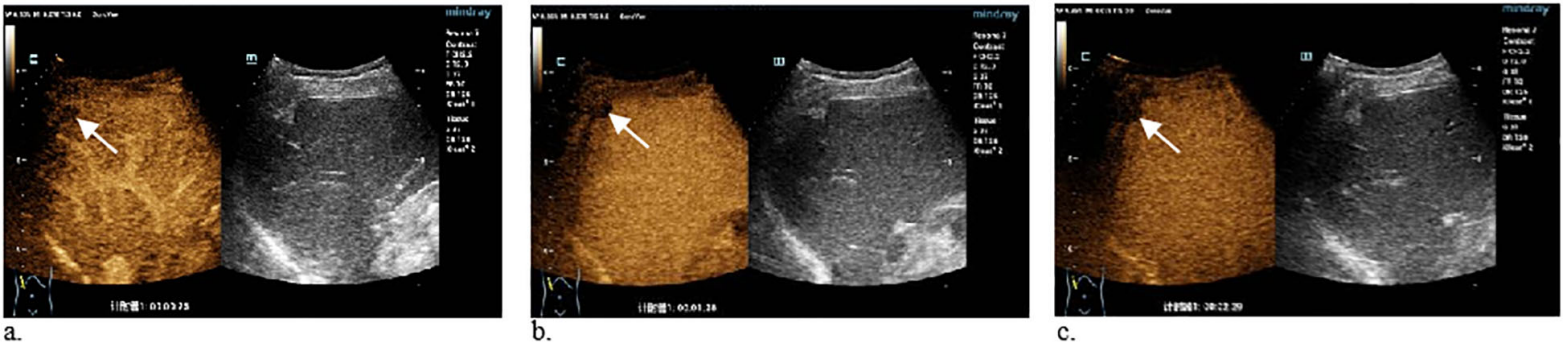
**(a–c)** CEUS images of the hepatic arterial, portal venous and parenchymal phases show no enhancement of the subperitoneal nodule in the right posterior lobe of the liver.

A 71-year-old man with constipation and irregular stools without abdominal pain was admitted to the hospital. There was a long history of alcohol consumption and smoking but no history of surgery. Pathologic findings of rectal biopsy at the local hospital suggested rectal adenocarcinoma. Preoperative abdominal CT at our hospital incidentally revealed the presence of a low-density nodule under the hepatic pericardium with calcification, which was diagnosed as a pseudolipoma (**Figure 1**). To further clarify the nature of the nodule, the patient underwent conventional ultrasound and contrast-enhanced ultrasound (CEUS) examination. Conventional ultrasound revealed a slightly hyperechoic nodule approximately 1.3x0.9 cm in size with poorly defined margins and a regular morphology under the pericardium of the right posterior lobe of the liver. (**Figure 2**). Doppler ultrasound revealed that the lesion was devoid of blood flow signals. After the injection of contrast agent with the patient’s consent, no enhancement was observed in the arterial, portal or parenchymal phases. The sonographer considered a benign lesion, possibly an isolated necrotic nodule or otherwise (**Figure 3**). On the basis of the patient’s clinical symptoms, test results, and imaging manifestations, the lesion was not considered a liver metastasis from rectal cancer. After three months of neoadjuvant chemotherapy, laparoscopic ultralow anterior resection of rectal cancer was performed. Symptomatic supportive therapy such as antibiotics to prevent infection and rehydration was subsequently administered. The patient recovered well and was discharged from the hospital. Postoperative and follow-up CT showed that the lesion was still present in the subperitoneum of the right lobe of the liver, with no change in morphology or size.

## Discussion

Glisson’s pseudolipoma is a concept originally proposed by Rolleston in 1891. This uncommon lesion is a considerable amount of degraded fat enveloped by the hepatic peritoneum, pathologically resembling an omental appendage and microscopically appearing as a thick hyaline fibrous capsule surrounding a cellular compartmental area of the stroma. The cells lack a nucleus and are composed of uniform basophilic material (5). It may migrate through the abdominal cavity according to the current literature, migration has occurred in only two cases, but it eventually settles on the diaphragmatic surface of the liver (6, 7). In this case, migration was not detected in the preoperative and postoperative CT scans, and no further follow-up was performed.

According to the reported radiological features, hepatic pseudolipoma presents on CT as a well-defined hypodense nodule with attenuation of fat or soft tissue in the center (8). The CT findings in this case were consistent with those described in the literature and included typical peripheral calcifications. Notably, in this report is that there was no previous report in the literature on the presentation of pseudolipoma after performing CEUS. This patient underwent CEUS and the lesion showed three periods of unenhanced presentation. This suggests that the nodule is a benign lesion but needs to be differentiated from an isolated necrotic nodule or other conditions. Solitary necrotic nodules are as rare as pseudolipoma, they have in common that they are both nodules that form after necrosis and are difficult to identify during surgery. Solitary necrotic nodules have been identified as intraparenchymal in the literature, which makes them clearly distinguishable. It tends to affect the right lobe of the liver and is situated in a peripheral location. Compared to the adjacent liver parenchyma, solitary necrotic nodules are hypodense without fatty attenuation and lack enhancement (9).

When pseudolipoma is detected, it needs to be differentiated from some other conditions. This patient had rectal adenocarcinoma, so it was necessary to distinguish pseudolipoma from metastasis (10). Pseudolipoma can easily be considered a metastatic lesion during open or laparoscopic surgery, especially if the patient has a malignant tumor of another organ. The presentation of metastatic hepatocellular carcinoma on grayscale US varies depending on the site of metastasis. Hypoechoic metastases are commonly observed, and hyperechoic metastases can also occur in tumors such as colorectal cancer. On CEUS, liver metastases can be hyperenhanced (hypervascular) or hypoenhanced (hypovascular). Metastases is characterized by rim-like enhancement and early and complete washout (11). This is very different from the CEUS mode used in this case. Distinguishing HCC from highly differentiated hepatocellular carcinoma (HCC) with fatty changes is not difficult. HCC is common in patients with hepatitis, especially cirrhosis, but hepatitis is also not detected in some patients. Grayscale US of HCC with fatty changes varies according to the size of the tumor and the blood supply and is characterized by complete enhancement and later washout on contrast. The presentation is usually independent of the fibrous envelope (12–14). This patient had no abdominal distension, abdominal pain, or other discomfort. There was no history of hepatitis B infection, and the alpha-fetoprotein level was within normal limits. Therefore, we do not consider the diagnosis of HCC.

Pseudolipoma in the liver are characterized by a thick fibrous envelope that clearly separates the lesion from the liver parenchyma (15). With this envelope, the lesion can be relatively easily differentiated from true lipoma and vascular smooth muscle lipoma. True lipomas of the liver are usually located within the hepatic parenchyma, have infiltrative margins, have no distinctive envelope, and are usually devoid of degenerative changes (16). Pseudolipomas are located outside of Glisson capsule, with an envelope separating the nodule from the hepatic parenchyma, and are associated with a distinct fibrous envelope and degenerative changes. This makes it easy to distinguish pseudolipoma from the equally rare true hepatic lipomas. In addition, it needs to be differentiated from benign liver lesions such as hepatic hemangiomas and focal fatty deposits. Patients with hepatic hemangiomas are usually asymptomatic, have no specific medical history, and are found only incidentally on some tests. This process is similar to that of pseudolipoma. Hepatic hemangiomas appear as well-defined hyperechoic or hypoechoic lesions on conventional ultrasound. On CEUS, hepatic hemangiomas are characterized by peripheral nodularity during arterial phase enhancement, followed by a centripetal filling (17). Focal fatty deposits routinely appear on ultrasound as a hyperechoic, nonspherical masses with slight posterior fat attenuation. On CEUS, the arterial, portal, and parenchymal phases show iso-enhancement with the liver parenchyma (18).

In conclusion, this case describes an elderly male with rectal adenocarcinoma whose pseudolipoma under the liver peritoneum on a preoperative abdominal CT scan. There is a lack of literature on hepatic pseudolipoma, and more is needed to report on the imaging manifestations of this disease, especially US and CEUS. CEUS is useful in the diagnosis of pseudolipoma, mainly for differentiating it from other benign and malignant lesions and may reduce the rate of misdiagnosis of benign liver lesions. CEUS is advantageous for identifying benign and malignant lesions in the liver and is able to visualize the imaging performance continuously after the injection of a microbubble contrast agent, revealing the enhancement process. The sensitivity of CEUS is similar to that of enhanced CT, but CEUS is a radiation-free examination, and the US contrast agent is not toxic to the liver or kidneys. However, laboratory tests and clinical manifestations should not be ignored when identifying lesions.

## Data Availability

The original contributions presented in the study are included in the article/**Supplementary Material**. Further inquiries can be directed to the corresponding author.
